# A Flexible and Low-Cost Tactile Sensor Produced by Screen Printing of Carbon Black/PVA Composite on Cellulose Paper

**DOI:** 10.3390/s20102908

**Published:** 2020-05-21

**Authors:** Yeter Sekertekin, Ibrahim Bozyel, Dincer Gokcen

**Affiliations:** 1 Deptartment of Electrical and Electronics Engineering, Hacettepe University, Ankara 06800, Turkey; yeter@ee.hacettepe.edu.tr (Y.S.); bozyel@ee.hacettepe.edu.tr (I.B.); 2 Deptartment of Nanotechnology and Nanomedicine, Hacettepe University, Ankara 06800, Turkey; 3 METU MEMS Research and Application Center, Ankara 06530, Turkey

**Keywords:** composite ink, impedance sensor, piezocapacitive, piezoresistive, screen-printing, paper sensor

## Abstract

This study presents the design and fabrication of a flexible tactile sensor printed on a cellulose paper substrate using a carbon black (CB) – filled polyvinyl alcohol (PVA) polymer matrix as ink material. In the design, electrodes are obtained by screen printing of CB/PVA composite on dielectric cellulose paper. The screen-printing method is preferred for fabrication because of its simplicity and low manufacturing cost. The tactile sensor is formed by overlapping two ink-printed sheets. Electrical properties are investigated under compressive and tensile strains. The results indicate that the tactile sensor configuration and materials can be used for piezoresistive, capacitive, and also impedance sensors. The same tactile sensor structure is also examined using a commercial carbon-based ink for performance comparison. The comparative study indicates that CB/PVA ink screen-printed on paper demonstrates superior sensitivity for capacitive sensing with low hysteresis, as well as low response and recovery times. The piezoresistive-sensing properties of CB/PVA on cellulose paper show a gauge factor (GF) of 10.68, which is also very promising when conventional metal strain gauges are considered. CB/PVA screen-printed on cellulose paper features impedance-sensing properties and is also sensitive to the measurement frequency. Therefore, the response type of the sensor can be altered with the frequency.

## 1. Introduction

Flexible strain sensors are of great interest in wearable electronics and electronic skin applications, not only because of their flexibility, but also their high sensitivity and simple process flow. Some of the applications for which strain sensors have gained attention include monitoring human health status [1,2,3,4], actualizing artificial intelligence [5], soft robotics [6,7], and human/machine interfaces [4,5,6,7,8]. Strain sensors detect changes in electrical properties, such as capacitance, resistance, or impedance, induced by applied mechanical stimulus. Depending on applications, they are also classified as tactile or pressure sensors. Tactile information generated by mechanical stimuli is collected by sensors and converted into electrical signals. Critical parameters for evaluating the performance and flexibility of strain sensors are precision, simplicity of structure, low cost, and flexibility. All these criteria highly depend on materials and process methods used for manufacturing. Sensor fabrication has gained momentum, and the importance of more effective materials in driving the sensor market is undeniable.

Extensive studies have been conducted in the literature on different methodologies to achieve higher sensitivity and flexibility, as well as low cost [6,8,9,10,11,12,13,14,15,16,17]. Carbon-based materials are strong candidates to be utilized in flexible sensors due to their unprecedented morphology and electrical and mechanical properties [10]. The most commonly used carbon nanomaterials for sensors are carbon black (CB), carbon nanotubes (CNT), and graphene [15]. CB has found a place in various applications of consumer products, such as pigments, plastics, sensors [16], and supercapacitors [18]. CB was used for flexible and wearable electronics due to its electrical and mechanical properties, as well as cost-effectiveness [16,19,20,21,22,23]. Graphene also is used for strain and electrochemical sensing. It has a 2D and honeycomb structure, and features such as ultra-translucency, high mechanical stability, and high restorability make graphene a good candidate for highly sensitive strain sensors [24]. Some studies realized using graphene include a strain sensor for monitoring human motion [24], pressure sensors [25], a stretchable sensor for strain, bending and torsion [26], and a stretchable as well as self-healing sensor [27]. Yan et al. proposed a piezoresistive graphene–cellulose paper for strain sensors to increase the sensing limit of graphene-based sensors [28]. They embedded the nanopapers made of crumpled graphene and nanocellulose into a stretchable elastomer matrix to manufacture a strain sensor. Qi and coworkers researched a mulberry-paper-based graphene strain sensor for wearable electronics [9]. They investigated the fabricated strain sensor in different aspects, such as flexibility, environmental stability and mechanical strength, and it was seen that the graphene strain sensor could be very suitable for wearable electronics. Another study that used CNT was realized by Khan et al. for the tactile sensors [29]. They compared multiwalled CNT (MWCNT)/polydimethylsiloxane (PDMS) composite to P (VDF-TrFE) sandwiched by silver layers. Hanbin et al. proposed a degradable paper-based strain sensor to detect human movements using an aqueous suspension of CB [14]. The proposed concept was not only degradable but also eco-friendly.

In addition to the design of materials used in sensors, establishing a process integration scheme for sensor fabrication is of great interest to obtain a high-quality and low-cost final product. Screen printing is one of the most widely used fabrication methods in wearable and flexible devices, because of the low cost, high yield, and uniform thickness over the surface [2]. Subramanya et al. fabricated disposable strain gauges using the screen printing method [11]. They used graphite as the carbon filler and glass as the substrate. The thickness of the screen-printed films varied in the range of 13–27 µm. They obtained easily manufactured, disposable, and robust strain gauges. One other study that used the screen-printing method for sensor fabrication was realized by Sinti et al. in 2017 [30]. The study aimed to detect ethanol in beer samples, using the screen-printed graphite electrodes modified by nanocomposite formed by CB and Prussian Blue nanoparticles on commercial paper. This was a facile and sustainable method for a disposable biosensor detecting ethanol.

This work explores the design and implementation of a CB/PVA composite ink-based flexible tactile sensor produced on a cellulose paper substrate. The sensor design relies on both the resistive and capacitive properties of the materials used. Here, a CB/PVA composite was used as the electrode material, whereas the dielectric material was the commercial cellulose paper. At the initial stage of this study, CB/PVA on cellulose paper was evaluated as a flexible piezocapacitive and piezoresistive strain sensor. Additionally, the produced CB/PVA composite was compared with a commercial ink to evaluate its performance as a flexible strain sensor. GF values obtained from piezoresistive sensitivity measurements imply the usability of CB/PVA ink printed on cellulose paper as a tactile sensor in wearable and flexible electronics. A screen-printing process was developed to pattern the structure on cellulose paper in the form of a tactile sensor. Capacitive and resistive measurements were conducted under different pressure values applied. Strain sensitivity, response, and recovery time measurements were carried out to investigate the performance of the CB/PVA composite and its interaction with the cellulose substrate. As compared to commercial conductive ink, the response and recovery times show better performances. Besides, the impedance properties of the designed sensor were observed under various frequency rates, and impedance variations were mapped in the sensor layout. To the best of our knowledge, the impedance characteristics of CB/PVA composite ink printed on cellulose paper was not examined previously. Impedance measurements combine piezoresistive and piezocapacitive properties in a single sensor configuration. Notably, the frequency dependence of a tactile sensor configuration represents a critical point of view to develop different measurement techniques while sensing. It is important to note that the response type of the impedance sensor can be adjusted via measurement frequency. The low-cost fabrication technique combined with sensitivity and responsivity emphasizes the potential of this work not only for the scientific community but also for the sensor and electronics industry.

## 2. Materials and Methods

### 2.1. CB/PVA Composite Ink Preparation

CB is formed of amorphous carbon nanoparticles with strong covalent bonds between each other. The preparation method for CB, such as oil furnace, gas furnace, the thermal process or the channel process [31], determines its particle size, surface area, and density. The CB particle diameter varies between 10 and 100 nm and surface area between 25 and 1500 m^2^ /g. The density is also lower than 2.25 g/cm^3^ [32]. It can be used as a nanofiller, such as graphite and CNT. While unfilled polymers are insulators, some conductive fillers with a polymer matrix may constitute conductive composites [32]. The resulting electron movement in the composite is known as percolation. CB is widely used as a conductive material in polymer matrixes and acts as a nanofiller in the composite [33]. The CB/polymer composites are widely used to fabricate flexible sensors. 

In this study, the ink used as the electrodes in the sensors was prepared in deionized water using CB and PVA. PVA (MW:85,000–124,000, Aldrich) is a water-soluble polymer and used as a polymer network in ink. PVA is preferred in this study due to its stretchable nature as well as easy solubility in water. It is biodegradable, biocompatible, hydrophilic, non-toxic, and low-cost [34]. The fact that PVA is suitable for 3D printing also makes it a preferred option due to the trend in printed sensors [35].

First, 6.28 g PVA in granular form was dissolved in 75 mL deionized water [34]. This procedure was carried out at 70 °C by stirring the mixture at 700 rpm for 30 h. Eventually, a gel form of the PVA was obtained from the granular form. In the following procedure, CB (Aldrich) was gradually added to the hot mixture to arrange the viscosity of the suspension for the screen-printing process. An ink having a suitable viscosity was attained as 2.182 gr CB was added to the gel and stirred for 2 h at 500 rpm. One of the points that should be emphasized is that the ink recipe is prepared to achieve suitable viscosity for screen printing as well as the desired electrical and mechanical properties. Table S1 also shows the electrical properties of ink materials prepared using different compositions of CB and PVA. Structural properties are critical parameters while determining the optimum ratio of CB to PVA. As seen in the figures in the Supplementary Material, as CB: PVA ratio increases, CB particles tend to form more massive clusters that do not present a tendency to be involved in the polymer matrix. 

### 2.2. Piezoresistive Sensing

Since commercial A4 cellulose papers are easily available and flexible, they were used as substrates to draw patterns on using CB/PVA-based ink. The resistance under strain can be expressed as Equation (1) [36].

(1) $$R=\frac{\rho }{\rho_{0}}R_{0}{\left(\varepsilon +1\right)}^{2}$$

where ρ and $\rho_{0}$ correspond to the electrical resistivity and the reference resistivity, respectively. $R_{0}$ is the resistance without tension strain, and ε is the tension strain. GF is used to evaluate the response and sensitivity of the strain sensor printed on a paper substrate in case of a mechanical stimulus. For piezoresistive sensors, the GF is found from the slope of the $\frac{\Delta R}{R_{0}}$ vs. ε graph. In other words, the GF is given by $\frac{\Delta R/R_{0}}{\varepsilon }$ [13], whereas ΔR is the change in the resistance. If the resistance equation in Equation (1) is substituted in the GF expression, Equation (2) is obtained.

(2) $$GF=\frac{1}{\varepsilon }\left\{\frac{\rho }{\rho_{0}}{\left(\varepsilon +1\right)}^{2}-1\right\}$$

### 2.3. Piezocapacitive Sensing

**Figure 1:**
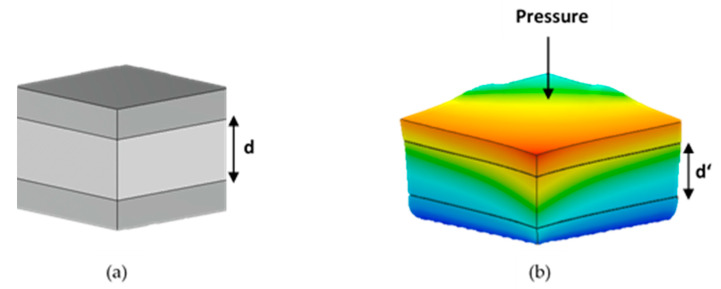
(**a**) Parallel plate capacitor. (**b**) Parallel plate capacitor under pressure.

A parallel plate capacitor configuration can be used to understand the sensing mechanism of a capacitive sensor. Figure 1a shows a simple structure of a parallel plate capacitor, and the capacitance is calculated by Equation (3). While *A* corresponds to the area of sensing electrodes, *d* is the distance between electrodes, which refers to the thickness of the dielectric material. $\varepsilon_{0}$ and $\varepsilon_{r}$ state the permittivity of free space and the relative permittivity of the dielectric material, respectively. When a pressure is applied, the initial thickness of the dielectric material, *d*, turns to *d’*, as can be seen in Figure 1b. This results in an increase in the capacitance.

(3) $$C=\frac{\varepsilon_{0}\varepsilon_{r}}{d}A$$

In this study, the cellulose paper substrate acts as a dielectric layer that is placed between the electrodes. Therefore, $\varepsilon_{r}$ expresses the dielectric constant for the cellulose paper. When the pressure is applied to the sensor, the dielectric material changes its formation, and the distance between sensing electrodes decreases. As can be seen in Equation (3), the capacitance increases with decreasing distance between electrodes. The capacitance value reaches saturation after a certain pressure value is reached because the distance between the electrodes cannot be reduced anymore.

### 2.4. Piezoimpedance Sensing

The impedance characteristics of the sensor were investigated to obtain capacitive and resistive effects at the same time under the applied pressure. The impedance expression in Equation (4) consists of real and imaginary parts corresponding to the resistance and the reactance, respectively. In addition, the phase angle represents the phase shift between the voltage and current signals and is calculated by Equation (5).

(4) Z=R+jX

(5) $$\theta =\tan^{-1}\left(\frac{X}{R}\right)$$

NI Elvis II+ was used as the impedance analyzer. The frequency range was varied between 200 Hz and 10 kHz. The impedance, resistance, reactance, and the phase angle were acquired by applying the pressure to each array of the tactile sensor configuration in Figure 4c. 

### 2.5. Surface Characterization of the Ink Material

Atomic force microscopy (AFM) (Nanomagnetics Instruments) was used three-dimensional (3D) investigation of the surface morphology of the produced CB/PVA ink on cellulose paper. AFM images were recorded in tapping mode due to the flexible structure of the polymer material. AFM provides information about the distribution, homogeneity, and 3D pattern of PVA containing CB. Scanning electron microscope (SEM) (FEI Quanta 200 FEG) was primarily used to visualize the surface structures of cellulose paper, PVA, and CB/PVA composite. SEM clearly provides an in-depth understanding of structural properties of coated materials and substrates.

### 2.6. Tactile Sensor Design

**Figure 2:**
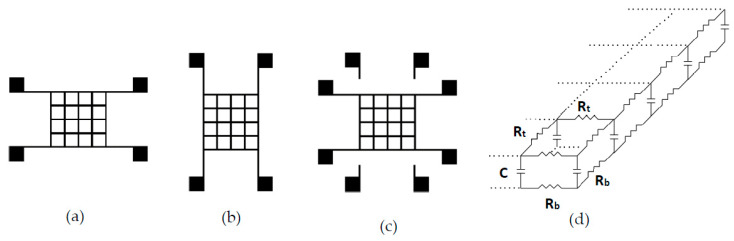
Illustrations of electrode patterns screen-printed on (**a**) top and (**b**) bottom sheets to produce sensor arrays, (**c**) tactile sensor produced by overlapped sensor arrays, and (**d**) equivalent circuit of the tactile sensor.

In this study, the tactile sensor is formed using two sensor arrays by placing one sensor array on top of the other one. The tactile sensor array to be fabricated is designed in a size of 4 cm × 4 cm (Figure 2a). Every square that consists of an array has an area of 1 cm^2^. Additionally, the pads extended from the array are used to measure electrical characteristics. One array was placed at the top; the other was placed at the bottom, as shown in Figure 2a,b, respectively. It is important to note that sheets were attached to each other using duct tape, and the bottom electrodes must contact the top sheet to ensure the reliability of the electrical contacts between the top and bottom arrays when pressure is applied. The attached structure was formed, as illustrated in Figure 2c. Here, in addition to the resistance from electrodes, a capacitive effect can be observed since cellulose paper acts as a dielectric layer. As shown in Figure 2d, C represents the capacitance between the sensors in the top and bottom layers, which refers to the capacitance of cellulose paper. While R_t_ represents the resistance of the ink material on the top sheet, R_b_ expresses the resistance of the ink material on the bottom sheet. When pressure is applied to the upper sensor, the measured resistance and capacitance values are both subject to changes; therefore, this setup prioritizes impedance values in sensing rather than only resistance or only capacitance values.

### 2.7. Screen-Printing Process

**Figure 3:**
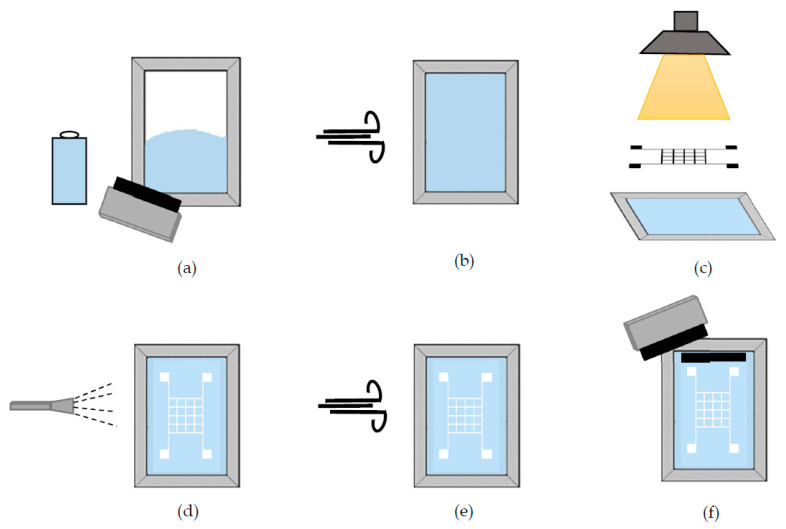
Screen-printing process flow (**a**) covering the mesh by photo emulsion (**b**) drying procedure (**c**) exposing to ultraviolet light (**d**) cleaning procedure (**e**) drying procedure (**f**) spreading of the ink using a squeegee.

The fabrication of each sensor array was realized using the screen-printing method. All stages in the screen-printing process flow are given in Figure 3. This method started by covering the front and back of the mesh by photo emulsion material (Diazo), as illustrated in Figure 3a. A mesh with 28-µm opening size and 180-cm count with 56-µm thickness was used in the process. The photo emulsion process enables the positive image of the mask to be transferred to the mesh. This process was done in a dark room, and the mesh was dried out before further processing (see Figure 3b). Following the photo emulsion process, the mask was placed on the mesh and exposed to ultraviolet light (see Figure 3c). The exposure time might be subject to changes according to the intensity of the light. The pattern on the mask was transferred to the mesh via the exposure. Then, cleaning (see Figure 3d) and drying (see Figure 3e) of the mesh were done. Before the last step, the cellulose paper was placed under the mesh. Eventually, CB/PVA composite ink was poured onto the mesh, and it was spread out on the mesh with the help of a squeegee to form the desired sensor array on the cellulose paper (see Figure 3f). The foremost reasons for using a paper substrate are that it is low-cost, easily available, and above all, make it relatively simple to produce sensors with feasible methods such as handwriting, printing, and screen printing. The polymeric substrates are generally hydrophobic and must be processed before the ink material can be applied to it [37]. The screen-printing methodology was also used for a carbon-based commercial ink, and its electrical performance was compared to the homemade ink.

### 2.8. Electrical and Mechanical Measurement Setup

**Figure 4:**
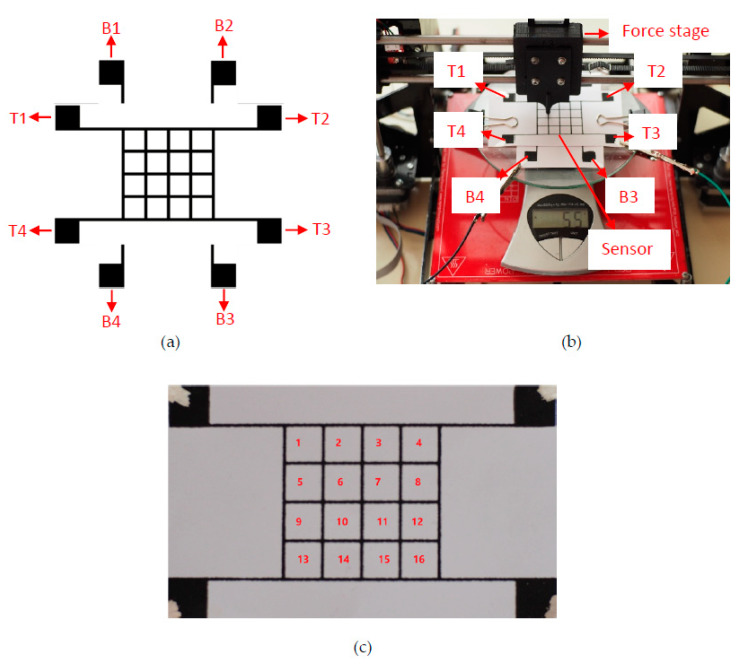
(**a**) Illustration of measurement pads of the tactile sensor. (**b**) Automated measurement setup capable of applying force and pressure at high precision. (**c**) The picture of a fabricated sensor array with numbers given to point the locations on the topmost sensor array.

As explained in the previous sections, the tactile sensor consists of two layers, and contacts at both layers are essential to take impedance measurements from the sensor. Figure 4a shows the labeling notation used for contacts. Here, T and B express the top and bottom layers, respectively. The impedance measurements were taken by placing the probes on a pair of pads, such as B1-T2, B1-T3, B1-T4, B2-T4, and B3-T4. Figure 4b demonstrates the automated force stage we designed to conduct electrical measurements while applying mechanical stimulus. Via this equipment, an equal amount of pressure can be applied to each point, and reliable measurements can be taken within a short time. The measured parameters include resistance, capacitance, impedance, and phase shift at various applied pressures and measurement frequencies. In the following, in order to refer to the locations where pressure was applied on the topmost layer, the labeling is shown in Figure 4c. As seen in the photo, a silver paste was applied to pads to conduct measurements. 

## 3. Results

### 3.1. CB/PVA Composite Ink on the Cellulose Paper

Before conducting detailed research on the tactile sensor design, we evaluated the electrical properties of CB/PVA composite ink on a cellulose paper as a simple flexible strain sensor. Initially, the CB/PVA composite ink was dried on the paper substrate to investigate its piezoresistive properties under compressive and tensile strain. As strain is applied, the resistance of the electrodes changes due to the fragmentation and realignment of CB aggregates [38]. 

**Figure 5:**
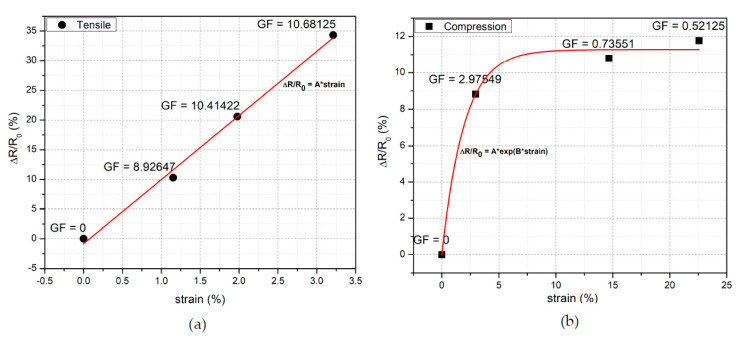
The relative change of the resistance versus (**a**) tensile strain and (**b**) compressive strain.

GF of the CB/PVA composite ink applied to cellulose paper was investigated under tensile and compressive strain. First, the initial length and resistance were measured; then, a digital caliper was used to adjust the length of the paper. Measurements were taken under the compression and tension of the cellulose paper by bending the paper in convex and concave directions, respectively. Each resistance value corresponding to measured length was recorded, and a graph was plotted using the relation between each of the initial and measured resistance and length values. In the case of a tensile strain, the relative change of resistance was increased up to 34% linearly, as can be seen in Figure 5a. GF is equal to the slope of the curve and measured as 10.68 under the maximum tensile strain (3.21%). The conventional metal strain gauges have a GF of nearly 2. Therefore, the measured GF shows promising results when compared to the conventional metal strain gauges, as well as some of the flexible [14] and stretchable [30] sensors worked out in the literature. 

In addition to the tensile strain, the GF was computed under the compressive strain. Figure 5b shows the change of resistance under the compressive strain. The change in the resistance was 8.8% under 3% compressive strain, and this corresponds to the GF of 2.97. If the compressive strain was increased above 3%, the relative change of resistance reached saturation, and the GF values were decreased. The compressive strain was increased up to 23%, and the mean of GF values was calculated as 0.6 in between 3% and 23% strain. The measurements indicate that the strain sensor formed by CB/PVA composite ink on cellulose paper has a higher sensitivity to the tensile strain. Therefore, the type of strain the sensor exposed to could be determined based on GF calculation. If the resistance changes abruptly with strain, and GF is relatively high, it means a tensile strain is applied to the sensor. Additionally, the relationship between resistance change and strain is linear under relatively low strain rates, regardless of the tensile or compressive strain.

The sensor made up of CB/PVA composite ink on cellulose paper is at rest position when no strain is applied. In this case, the carbon particles are connected to each other and form a reasonably conductive path along the electrode material. However, if the sensor is subjected to compressive or tensile stress, an interruption in the carbon network begins, and, as the applied tension increases, the fragmentation in the carbon bonds increases. This causes increased resistance due to the reduced number of conductive paths. When the applied strain is released, the carbon particles in the network are reformed in the rest position, and the discontinuity in the conductive lanes is eliminated.

**Figure 6:**
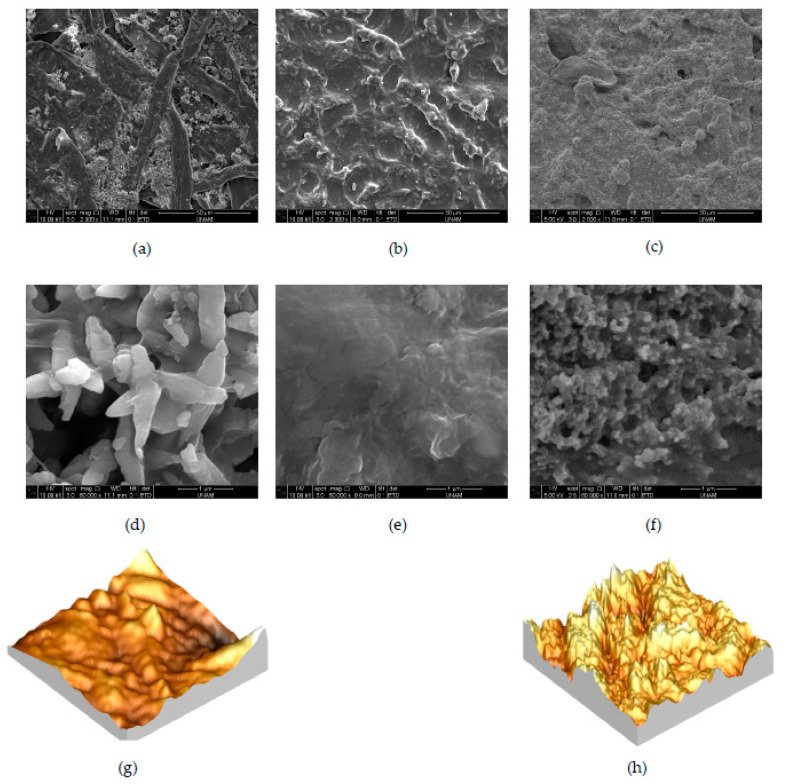
SEM images of (**a**–**d**) cellulose paper (**b**–**e**) PVA (**c**–**f**) CB/PVA composite on cellulose paper, and 3D AFM images of (**g**) the blank cellulose paper (image dimensions 5 μm × 5 μm) and (**h**) the CB/PVA composite ink screen-printed on cellulose paper (image dimensions 10 μm × 10 μm).

SEM images in Figure 6 are obtained at different magnifications (Figure 6a–c: ×2000, Figure 6d–f: ×60,000). Figure 6a,d show the microstructure of pure cellulose paper. It is clearly seen that cellulose paper has a fibrous structure, and PVA paste applied to the paper fills gaps between fibrous structures, as shown in Figure 6b,e. SEM images of CB/PVA composite ink screen-printed on cellulose paper are shown in Figure 6c,f. As can be seen in the figures, the surface structures of cellulose paper, PVA, and CB/PVA do not resemble each other, and each material has its own signature on the surface. As Figure 6e,f are compared, agglomeration of CB nanoparticles within PVA can be clearly observed. In other words, Figure 6f exhibits nanoporous structures formed by the addition of CB nanoparticles into the PVA complex. As discussed in the previous paragraph, the presence of CB nanoparticles and their arrangement in the composite structure is critical to obtain the desired sensitivity, responsivity, and conductivity, as well as mechanical properties [27]. Consistent with the literature, the size of CB nanoparticles is found to be around 100 nm from SEM images [25]. 

Figure 6g,h show representative 3D AFM images obtained from CB/PVA composite screen-printed and blank cellulose papers. In agreement with SEM images, high-resolution AFM images show that CB particles were distributed all over polymer chains, forming clustered structures. The non-uniform distribution of the ink material highly depends on the non-uniform surface structure of the commercial paper. Root-mean-square (RMS) roughness is an indication of surface morphology. According to the statistical analysis of AFM images recorded on CB/PVA composite on cellulose paper and blank cellulose paper, CB/PVA printed surface (RMS roughness = 56.8 nm) demonstrates lower roughness than blank paper (RMS roughness = 72.4 nm). As mentioned during the discussion of SEM images, PVA fills gaps in fibrous cellulose paper, and roughness decreases with the filling of the fibrous structure.

Additionally, a comparison of piezoresistive properties of our sensor material with other materials reported in the literature is given in Table 1 for GF and applied maximum strain. Accordingly, the sensor produced by screen printing of CB/PVA composite on cellulose paper provides higher GF compared to many relevant studies. Especially when the fabrication procedure and performance parameters are evaluated together, CB/PVA composite on cellulose paper has the potential to be utilized in flexible and wearable electronic systems.

### 3.2. Tactile Sensor

In this section, all sensor structures discussed refer to tactile sensors given with the geometry in Figure 2a. Sensors produced using the commercial carbon-based ink and homemade CB/PVA composite ink were compared from different perspectives. The piezoresistive properties of ink material were detailed in the previous section, and, here, due to the dielectric nature of cellulose paper, we will mostly discuss capacitive and impedance characteristics for sensing properties of the tactile sensor. In addition to the electrical properties, the response and recovery times of the tactile sensors were also evaluated.

#### 3.2.1. Piezocapacitance Characteristics of the Tactile Sensor

**Figure 7:**
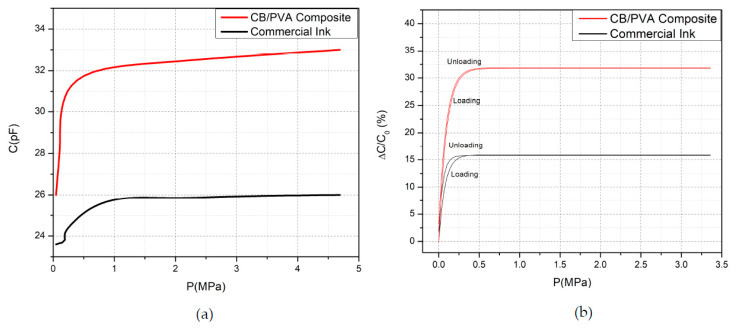
(**a**) Capacitance–pressure characteristics of the sensors. (**b**) Hysteresis behavior.

Figure 7a shows the relationship between the capacitance and applied pressure on the sensors. The pressure was applied to the mid-point of the arrays labeled 2, 3, 6, and 7 in Figure 4c, and measurements were recorded by probing T1-B3 (see Figure 4a). Accordingly, the CB/PVA composite ink-based tactile sensor presents higher capacitance values than the commercial carbon-based sensor when the pressure is applied at the rest position. The sensitivity of the pressure sensing is described by the slope of the line, and this is mostly used to evaluate the performance of pressure sensors [44]. As can be seen in Table 2, the sensitivities of sensors were examined at three different pressure ranges. The commercial ink-based sensor has a sensitivity of 0.15 MPa^−1^ at the pressure range of 0–312 kPa. The sensitivity of the CB/PVA composite-based ink is 1.595 MPa^−1^ at the range of 0–156 kPa. This indicates that the most effective pressure range, at which the highest sensitivity was achieved, was 0–156 kPa. The sensitivity can be compared with some of the previously presented studies, such as sensors made up of silver nanowire on Ecoflex (1.62 MPa^−1^ for below 500 kPa) [45], and gold thin film embedded on silicone rubber (0.4 MPa^−1^ for up to 160 kPa) [46]. Apparently, the sensor utilizing CB/PVA composite-based ink outperforms the sensor using the commercial ink, and this may originate from carbon and PVA content in the ink material. Conductive paths established by CB particles are positioned on PVA chains, and PVA is a dielectric material. Therefore, in addition to the capacitive properties of the cellulose paper, PVA induces extra capacitance. Cellulose paper can be compressed on a small scale owing to its fibrous structure. Different types of papers can be used to improve compressibility [20]. In this study, the presence of PVA in the electrode material promotes compressibility significantly via its stretchable nature. The compression of both ink materials and cellulose paper provide noteworthy improvements in the capacitive sensing. The CB/PVA composite ink-based sensor also reaches the saturation value at higher pressure rates. When the pressure is applied to the sensor and then released, the viscoelasticity of the polymer causes the sensor to have different capacitance values as compared to the initial state. This implies the presence of hysteresis in the measurements, which is crucial for dynamic loading of the strain sensors. Wide hysteresis causes irreversible effects on the sensors, and the capacitive sensors exhibit better hysteresis performance than resistive sensors [47]. Figure 7b shows the hysteresis behavior of the sensors which were fabricated by using both CB/PVA composite and commercial inks. Both sensors have good hysteresis behaviors. The CB/PVA composite ink-based sensor has a larger capacitance value, and its hysteresis is relatively narrower than the commercial ink-based sensor. 

Besides the pressure-sensing characteristics of the sensors, their touch-sensing properties were evaluated. Capacitance change with the touch of a finger is caused by the deterioration of the fringing electric field [45]. When a properly grounded conductor, or a finger in this experiment, touches the sensing electrodes, the fringing electric field over the capacitor is partially blocked by the finger, and thus the capacitance is reduced [45,46]. However, if the capacitor is touched by applying a force with the finger, two effects occur on the sensor: (1) as a grounded conductor, the finger reduces the capacitance; (2) the applied pressure force reduces the thickness of the dielectric material and thus increases the capacitance. Table 3 shows capacitance values observed at the touch of a finger with forces varying from 0.1 N to 10 N. While initial capacitance values of the sensors under conditions at which no force applied are close to each other, the difference becomes more observable as greater force is applied. One crucial point is that the CB/PVA-based ink reaches to saturation at greater levels of force and larger capacitance values. This can be correlated with the stretchable nature of PVA.

#### 3.2.2. Piezoimpedance Characteristics of the Tactile Sensor

**Figure 8:**
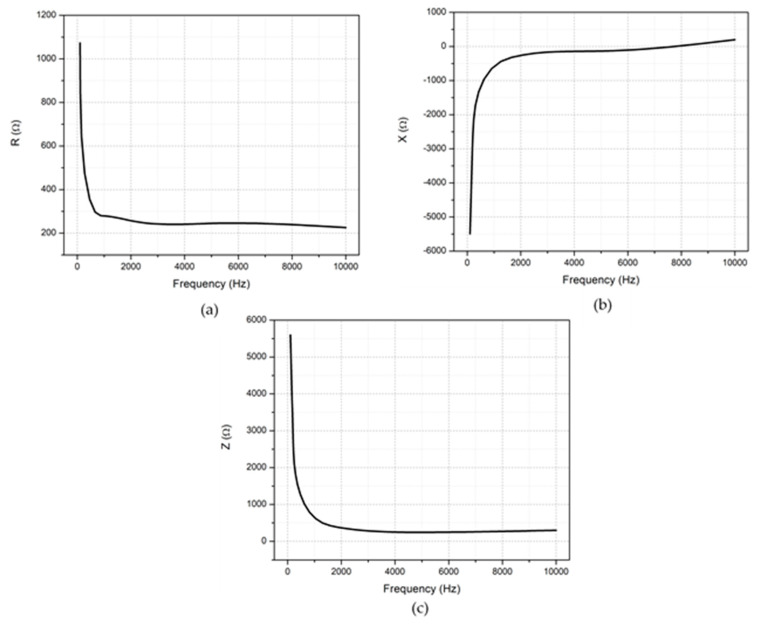
The impedance characteristics of the tactile sensor (**a**) the resistance (R) (**b**) the reactance (X), and (**c**) the impedance (Z) vs. frequency.

Similar to the piezocapacitive measurements, for piezoimpedance measurements, the pressure was applied to the mid-point of arrays labeled as 2, 3, 6, and 7 in Figure 4c, and the measurements were recorded by probing B3-T1 (see Figure 4a). An NI Elvis II+ impedance analyzer allows measuring impedance parameters at various frequencies using different pairs of measurement pads. Measurement frequencies were swept from 200 Hz to 10kHz. Figure 8a shows how the increase in frequency changes the resistance. As seen in the figure, the resistance decreases with increasing frequency. This feature could be explained by Jonscher’s power law, which, as given in Equation (6), expresses the relation between AC conductivity ($\sigma_{ac}$) and frequency (*f*) [48].

(6) $$\sigma_{ac}=\sigma_{dc}+A{\left(2\pi f\right)}^{n}$$

where $\sigma_{dc}$ is DC conductivity, A is the pre-exponential factor, and n is the fractional exponent (0 < *n* < 1). Equation (6) states that increasing the frequency leads to the raising of conductivity. Since the conductivity is inversely proportional to the resistivity (i.e., σ=1/ρ), increasing the frequency drives the decrease of the resistance. Figure 8b shows the relationship between the reactance and the frequency. The magnitude of the reactance decreases as the frequency increases up to nearly 7.5 kHz, which indicates that the capacitive response of the sensor becomes less effective at increasing frequencies. The capacitive reactance (*X_c_*), which is the imaginary part of the impedance, is expressed in Equation (7). Since the measurements were executed under constant pressure, the capacitive reactance change was only monitored for the changing frequency. Eventually, the capacitive reactance decreases at higher frequencies.

(7) $$X_{c}=\frac{1}{2\pi fC}$$

The sensor shows an inductive property after the measurement frequency of 7.5 kHz. The impedance response to the increasing frequency is given in Figure 8c. In a similar trend with the changes in the resistance and reactance, the total impedance decreases as the frequency increases, also implying a raise of the phase angle. It can be seen that the impedance approaches the resistance at high frequencies. Eventually, the measurements reveal that the sensor has a response to the frequency change under constant pressure.

Within the scope of this study, maps showing the impedance change distribution over the sensor were obtained when pressure was applied to specific locations of the tactile sensor. As the applied pressure increases on the specific location of the sensor, the reactance, resistance, as well as impedance values decrease. An increase in the pressure raises the capacitance value, and it also increases the conductivity by lessening the distance between CB particles. Therefore, the impedance distribution maps were used to locate the touchpoint. For this purpose, the impedance distribution was measured from different measurement pads, i.e., B1-T2, B1-T3, B1-T4, B2-T3, B2-T4 and B3-T4 as given before (see Figure 4). When the pressure was applied to a specific point, the impedance variation was calculated by comparing the simultaneously measured value with the one recorded at the rest position. As seen in the sensor structure, each location is surrounded by four lines (electrode). The pressure was applied to four symmetrical points close to each surrounding line, and the impedance values were compared to the initial ones to obtain a distribution map. The contour plot of four measurement points was used to display the absolute value of the impedance change in the specific location.

The point at which the pressure was applied can be located via electrical measurements using several pads. As provided in the experimental procedures, the setup in Figure 4b was used with an impedance analyzer to conduct the required measurements. The force stage, managed by a microcontroller, was prompted to apply 0.5 N (~780 kPa) at a constant frequency of 200 Hz. The impedance analysis was performed for the specific point where the pressure was applied using each pair of measurement pads (B1-T2, B1-T3, B1-T4, B2-T3, B2-T4, B3-T4). The results were analyzed and compared using LabVIEW software. Accordingly, each specific touchpoint can be located using corresponding measurement pads that will be detailed with examples in the following text. 

**Figure 9:**
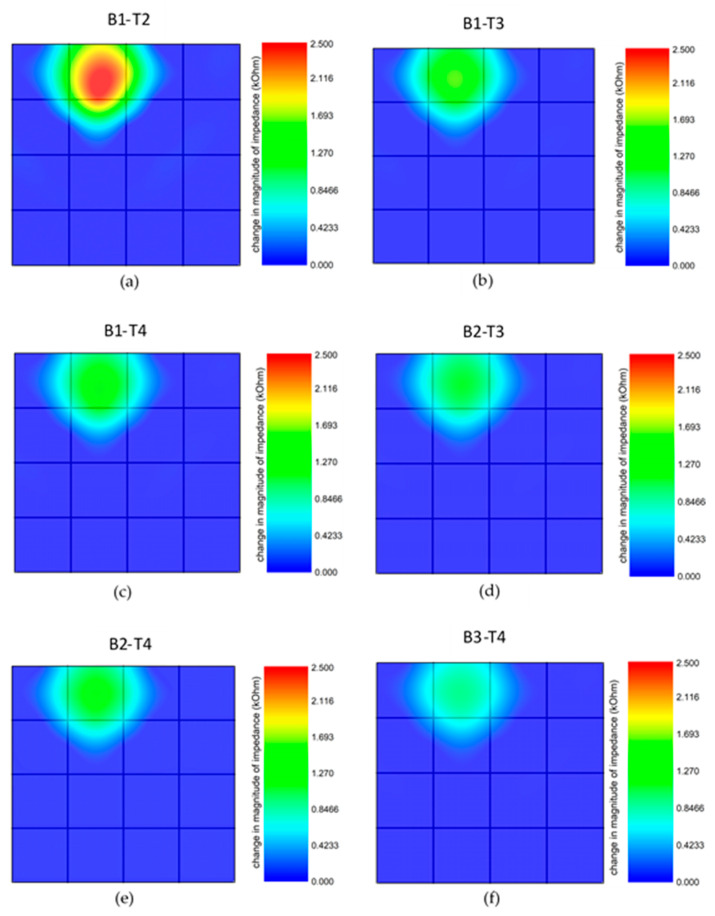
(**a**–**f**) The impedance distribution maps of the tactile sensor for the pressure applied on the specific region (labeled as 2 in Figure 4c) of the array obtained using different measurements.

Figure 9 exhibits variations in impedance measurements when different measurement pads were used. In this particular example, the pressure was applied to the point labeled 2 in Figure 4c. If a specific touchpoint is taken into consideration, the measured impedance value highly depends on the selected measurement pads. Thus, each point on the sensor can be sensed effectively using the predetermined pads. In the given sensor structure, the probes placed on the pads measure the impedance, which truly refers to effective resistance for an AC signal. The minimum resistance measured by an impedance analyzer or LCR meter along possible paths between two probes is actually the effective resistance. The screen-printing method provides a fairly uniform distribution of ink material over the paper, meaning that the resistance of each line with the same length is almost equal to each other. With this supporting information, the measurement pads can be randomly selected to locate the point or array where the force is applied. For the precision in the measurement, the touch should be at a point in between two measurement pads. Figure 9 shows the variation in the impedance measurement depending on the pair of measurement pads. Here, the force is applied to the area labeled as number 2 (red region in Figure 9a) on the sensor array, and the highest impedance magnitude was 2.5 kΩ at 200 Hz, measured using pads B1 and T2. When the measurements from each pair of pads are compared, B1-T2 pads reveal the change in the impedance value, with a higher magnitude for the specific touchpoint. While B1-T3 and B1-T4 exhibit close magnitudes, the minimum impedance change is observed using B3-T4 pads, as seen in Figure 9f. B3-T4 pads are the farthest from the touchpoint.

**Figure 10:**
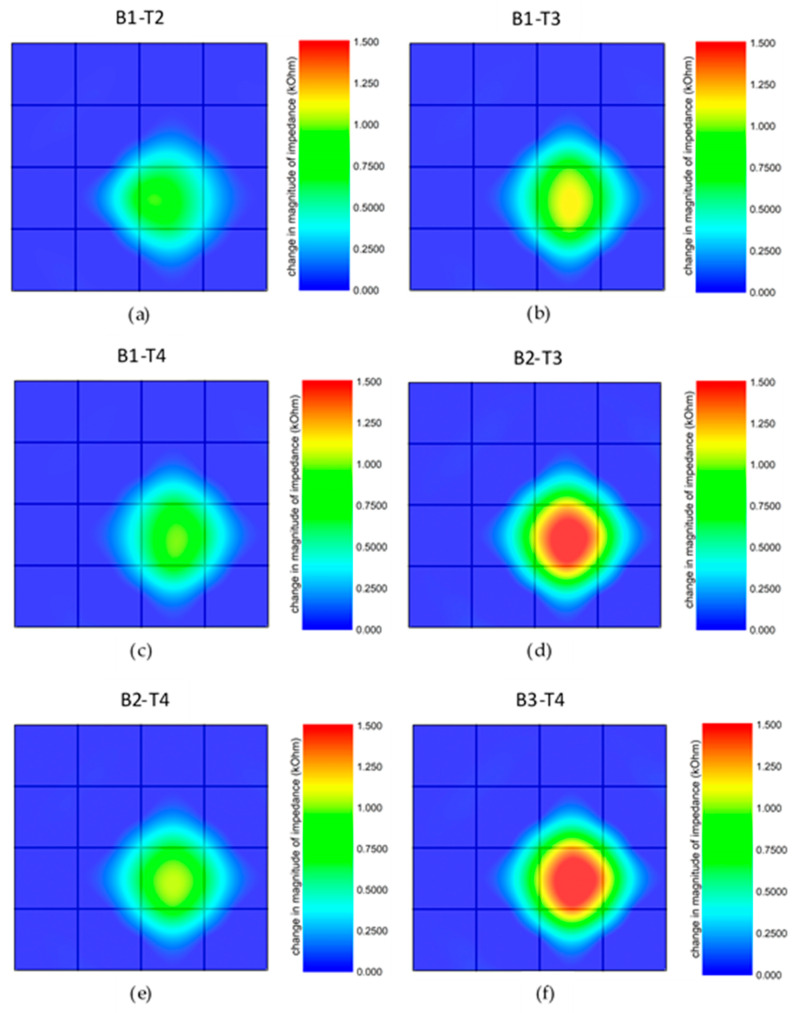
(**a**–**f**) The impedance distribution maps of the tactile sensor for the pressure applied on the specific region (labeled as 11 in Figure 4c) of the array obtained using different measurement pads.

Figure 10 shows the relation between the pair of probing pads and the change in the impedance value for the touchpoint labeled as 11 in Figure 4c. Once the region is touched with 0.5 N, the highest impedance magnitude was measured as 1.5 kΩ at 200 Hz using pads B2 and T3. In this case, the touchpoint has the same distance to particular measurement pads. For instance, B2-T3 and B3-T4 pads, as well as B1-T2 and B1-T4, are symmetrical to the point of interest. When the pressure is applied to the red region in Figure 10, it is expected to measure the same impedance value from the symmetrical measurement pads when the touchpoint is considered as the origin. B1-T2 and B1-T4, as well as B2-T3 and B3-T4, demonstrate the identical impedance distribution. The different magnitude values for the pressure applied to the specific point affects the minimum path resistance between pads. The equivalent circuit used to simulate the sensor structure is provided in Figure S5. In the figure, the pressure applied to a specific location is modeled via variations in resistance and capacitance values. As the pressure is applied, the resistance decreases, and the capacitance increases compared to the initial values. Table S2 demonstrates the relationship between the location where the pressure is applied and the change in the current values observed via different pair of measurement pads. 

In a typical resistive tactile sensor, three measurement points could be sufficient to position the touch of a finger. On the other hand, each array and column of the sensor array is subject to measurements in the capacitive tactile sensors, and this provides high precision to locate the point where the force is applied. It is important to note that the response of the CB/PVA composite ink-based sensor can change with the measurement frequency, as mentioned before.

### 3.3. Response and Recovery Time of the Tactile Sensor

**Figure 11:**
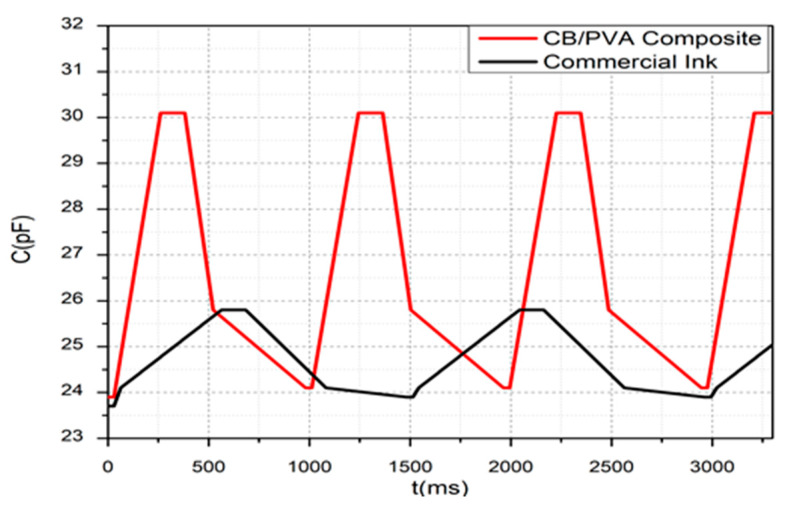
The capacitance-time characteristic under a specific load.

Response and recovery times are among the critical evaluation parameters for strain sensors. The term response time is used to determine when the sensor reaches a steady-state response, while the recovery time indicates how quickly the sensor returns to its initial state [33]. In this study, we applied a pressure of 100 kPa on an array of the tactile sensor and measured the change in capacitance over time, as shown in Figure 11. The results show that the response time of the CB/PVA composite based sensor is approximately 230 ms, and the recovery time is almost 600 ms. The reason for the more extended recovery period is the polymer structure changes after the pressure is applied. In other words, the friction force between fillers and polymer matrices causes a slow recovery [33]. Instrumental delays caused by measurement devices should be considered during these measurements. As seen in Figure 11, the same tests were performed on the tactile sensor using commercial ink as electrode material. Accordingly, the response time is 530 ms, and the recovery time is about 1 s. The response time of the CB/PVA composite based sensor is comparable to the stretchable sensors reported in the literature [12,14]. 

## 4. Conclusions

In this study, fabrication of a low-cost tactile sensor was achieved via screen printing of CB/PVA composite ink on cellulose paper. The piezocapacitive properties under the applied pressure, the strain sensitivity, the response and recovery times were examined, and the performance outcomes of CB/PVA composite was compared with those of a commercial conductive ink. Particularly, impedance-sensing performance was evaluated since both resistive and capacitive sensing properties were promising. It can be concluded that the CB/PVA composite-ink-based sensor has a piezoimpedance characteristic, and, therefore, the response type of the sensor can be changed with the frequency. The sensitivity of the CB/PVA-based sensor is 1.595 MPa^−1^, up to the applied pressure of 0.156 MPa. Additionally, the strain sensitivity was determined by the GF, which was computed as 10.68 under the tensile strain. The response and recovery times were obtained as 232 ms and nearly 600 ms, respectively. These results show that the fabricated sensor using CB/PVA composite can be used as a strain sensor as well as a tactile sensor. In addition, the quick response to the change of the frequency implies that the sensor may be used for fast-tracking of the touch of a finger.

## Figures and Tables

**Table 1:** Comparison of piezoresistive sensors in the literature.

Ref	Sensing Principle	Fabrication Method	Materials	GF (in Tension)	Max Strain (%)
[9]	piezoresistive	meyer-rod coating	mulberry paper/graphene	3.82	0.6
[14]	piezoresistive	dip coating	paper/CB	4.3	0.6
[39]	piezoresistive	pyrolysis	carbon paper/PDMS	25.3	20
[36]	piezoresistive	drop casting	CB/ecoflex	1.62–3.37	50
[19]	piezoresistive	self-assembly	polyethylenimine-reduced graphene oxide (PEI-rGO)/PDMS	1744	5
[20]	piezoresistive	drop casting	CB/PVA/airlaid paper	-	60
[40]	piezoresistive	screen-printing	MWCNT/epoxy	7.81–11.65	1.1
[41]	piezoresistive	ink-jet printing	graphene/paper	125	1.25
[42]	piezoresistive	drop casting	polyethylene terephthalate (PET)/graphene	0.11	7.5
[43]	piezoresistive	spin coating	functionalized graphene nanoplatelets(f-GNPs)/PDMS	3	0.2
this study	piezoresistive	screen-printing	CB/PVA/cellulose paper	10.68	3.4

**Table 2:** Pressure sensitivity of the sensors in different pressure ranges.

	Pressure Range	Sensitivity
Commercial ink-based sensor	0–0.312 MPa	0.15 MPa^−1^
	0.312–1.953 MPa	0.032 MPa^−1^
	1.953–4.687 MPa	2.33 × 10^−3^ MPa^−1^
CB/PVA composite ink-based sensor	0–0.156 MPa	1.595 MPa^−1^
	0.156–0.718 MPa	0.118 MPa^−1^
	0.718–4.687 MPa	0.010 MPa^−1^

**Table 3:** Capacitance values observed at the touch of a finger with different forces.

	Capacitance (pF)	
F (N)	CB/PVA Composite Ink-Based Sensor (C_0_ = 24.8)	Commercial Ink-Based Sensor (C_0_ = 23.5)
0.1	43.5	36.1
0.3	44.8	37
0.6	45.6	38.1
0.95	45.98	38.9
1.5	46.8	40.9
10	47	40.92

## Supplementary Materials

The following are available online at https://www.mdpi.com/1424-8220/20/10/2908/s1, Table S1: The impedance variation of the composite depending on CB/PVA ratio and frequency, Table S2: Color map showing the change in the current as the specified location is pressed. (Locations are labeled in Figure 4c.), Figure S1: (a)–(b) Optical images of CB/PVA composite with ratio of 0.2, Figure S2: Optical images of CB/PVA composite with ratio of 0.2. Exposing visible light from the blank side of the cellulose paper reveals that there are no macro-scale defects observable on the ink material, Figure S3: Optical images of CB/PVA composite with ratio of 1.2. Clustering of CB particles is observable even with bare eye, Figure S4: CB/PVA composite with ratio of 1.2. Exposing visible light from the blank side of the cellulose paper reveals the non-uniformity, as well as pores in the ink material. Figure S5: The equivalent circuit of the sensor structure (extended version of the equivalent circuit given in Figure 2d). The simulation is realized for a 5V, 200 Hz signal. The pressure is applied to Location #16 (labeled in Figure 4c in the article).
