# Supplementary material for: A Flexible and Low-Cost Tactile Sensor Produced by Screen Printing of Carbon Black/PVA Composite on Cellulose Paper
Source: Sensors (Basel). 2020 May 21;20(10):2908. doi: 10.3390/s20102908 (PMC7285113; doi:10.3390/s20102908)
Supplement: Supplementary file 1 [file sensors-20-02908-s001.pdf]

SUPPLEMENTARY MATERIAL

A Flexible and Low-Cost Tactile Sensor Produced by Screen Printing of Carbon Black/PVA Composite on Cellulose Paper

Yeter Sekertekin [1], Ibrahim Bozyel [1] and Dincer Gokcen [1,2,3,*]

[1] Dept. of Electrical and Electronics Engineering, Hacettepe University, 06800 Ankara, Turkey; yeter@ee.hacettepe.edu.tr (Y.S.); bozyel@ee.hacettepe.edu.tr (I.B.)
[2] Dept. of Nanotechnology and Nanomedicine, Hacettepe University, 06800 Ankara, Turkey;
[3] METU MEMS Research and Application Center, 06530 Ankara, Turkey;
* Correspondence: dgokcen@hacettepe.edu.tr

Received: 18 April 2020; Accepted: 18 May 2020; Published date:

Table 1. The impedance variation of the composite depending on CB/PVA mass ratio and frequency.

CB/PVA Mass Ratio	Impedance Magnitudes for Different Frequency Values				
	100 Hz	120Hz	1kHz	10kHz	100kHz
0.03	2.762 kΩ	2.761 kΩ	2.762kΩ	2.76kΩ	2.757kΩ
0.2	167.4Ω	167.43Ω	167.44Ω	167.44Ω	167.44Ω
1.2	1.118kΩ	1.166kΩ	1.1162kΩ	1.1125kΩ	1.109kΩ
This study (0.35)	531Ω	527.5Ω	527.2Ω	527.1Ω	526.9Ω

Table S1 shows how CB/PVA mass ratio affects the electrical characteristics of the composite at various frequency rates. When the amount of CB particles is less than PVA in the composite ink (Ratio=0.03), the resistance is higher. As the CB particles in the ink increase (Ratio=0.2), the resistance decreases.

Figures S1 and S3 show optical images of the ink materials with different compositions printed on cellulose paper. As seen in Figure S2, exposing visible light from the blank side of the cellulose paper reveals that there are no visible defects observable on the ink material. If CB particles are more abundant than PVA (Ratio=1.2, see Figure S3), the conductivity decreases due to the agglomeration of CB in PVA. This phenomenon is clearly seen in Figure S4. Additionally, exposing visible light from the blank side of the cellulose paper reveals the non-uniformity, as well as pores in the ink material made with the CB/PVA ratio of 1.2.

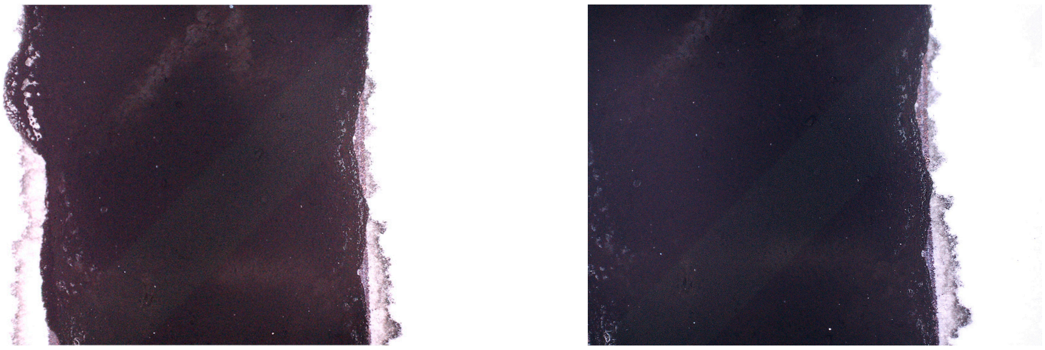

(a) (b)

Figure S1. (**a**) – (**b**) Optical images of CB/PVA composite with ratio of 0.2.

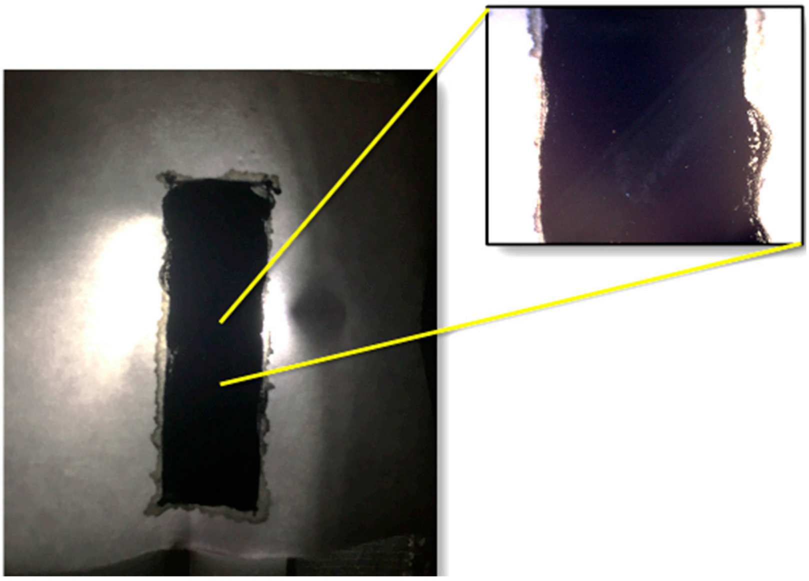

Figure S2. Optical image of CB/PVA composite with ratio of 0.2. Exposing visible light from the blank side of the cellulose paper reveals that there are no macro-scale defects observable on the ink material.

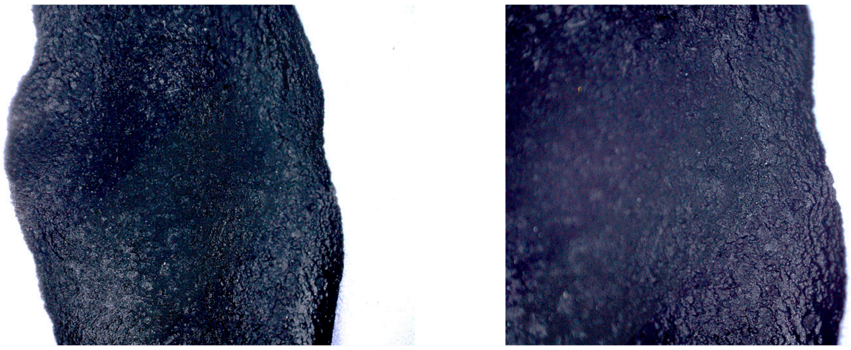

(a) (b)

Figure S3. (**a**) – (**b**) Optical images of CB/PVA composite with ratio of 1.2. Clustering of CB particles is observable even to the bare eye.

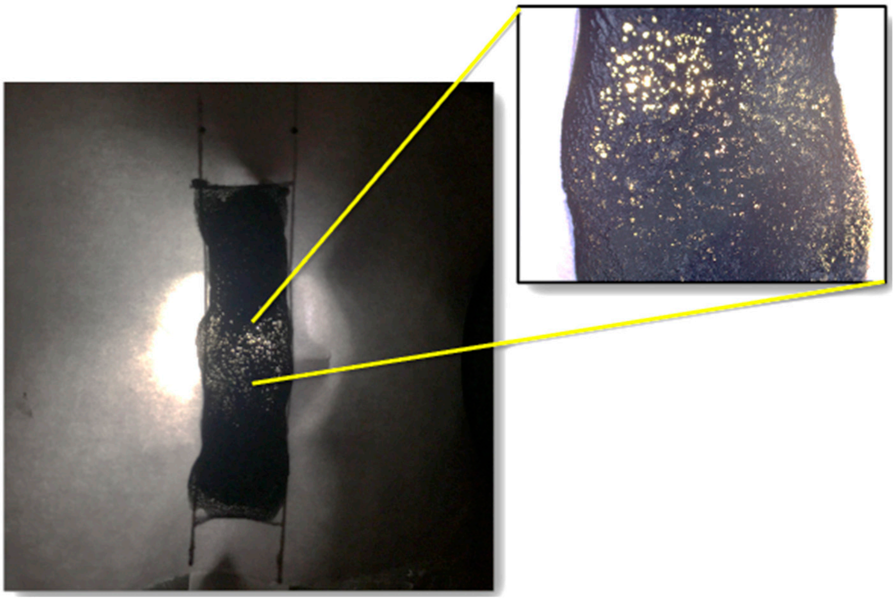

Figure S4. CB/PVA composite with ratio of 1.2. Exposing visible light from the blank side of the cellulose paper reveals the non-uniformity, as well as pores in the ink material.

Table S2 illustrates the lookup table used in the home-made software to locate the touchpoint in the form of a color map. To build the color map, impedance values were measured in the rest condition and the case when pressure was applied. Ohm's law states the relationship between the impedance and current (i.e. $V = ZI$). Each color and letter refer to a different value of current change, and so of impedance change under a constant voltage (5V, 200 Hz). In addition to experimental studies, the color map was also confirmed via simulation studies by the representation of electrodes and cellulose paper with different capacitance and resistance values. Figure S5 shows the schematic of the equivalent circuit of the sensor structure used in simulation studies. In this example, the pressure was applied to location #16 (Figure 4c.)

Table S2. Color map showing the change in the current as the specified location is pressed. (Locations are labeled in Figure 4c.).

Location #	Measurement Pads					
	B1-T2	B1-T3	B1-T4	B2-T3	B2-T4	B3-T4
1	A	B	A	C	D	C
2	G	F	E	H	I	J
3	G	I	H	E	F	J
4	A	D	C	A	B	C
5	E	F	G	J	I	H
6	K	L	K	M	N	M
7	K	N	M	K	L	M
8	E	I	J	G	F	H
9	H	I	G	J	F	E
10	M	N	K	M	L	K
11	M	L	M	K	N	K
12	H	F	J	G	I	E
13	C	D	A	C	B	A
14	J	I	E	H	F	G
15	J	F	H	E	I	G
16	C	B	C	A	D	A

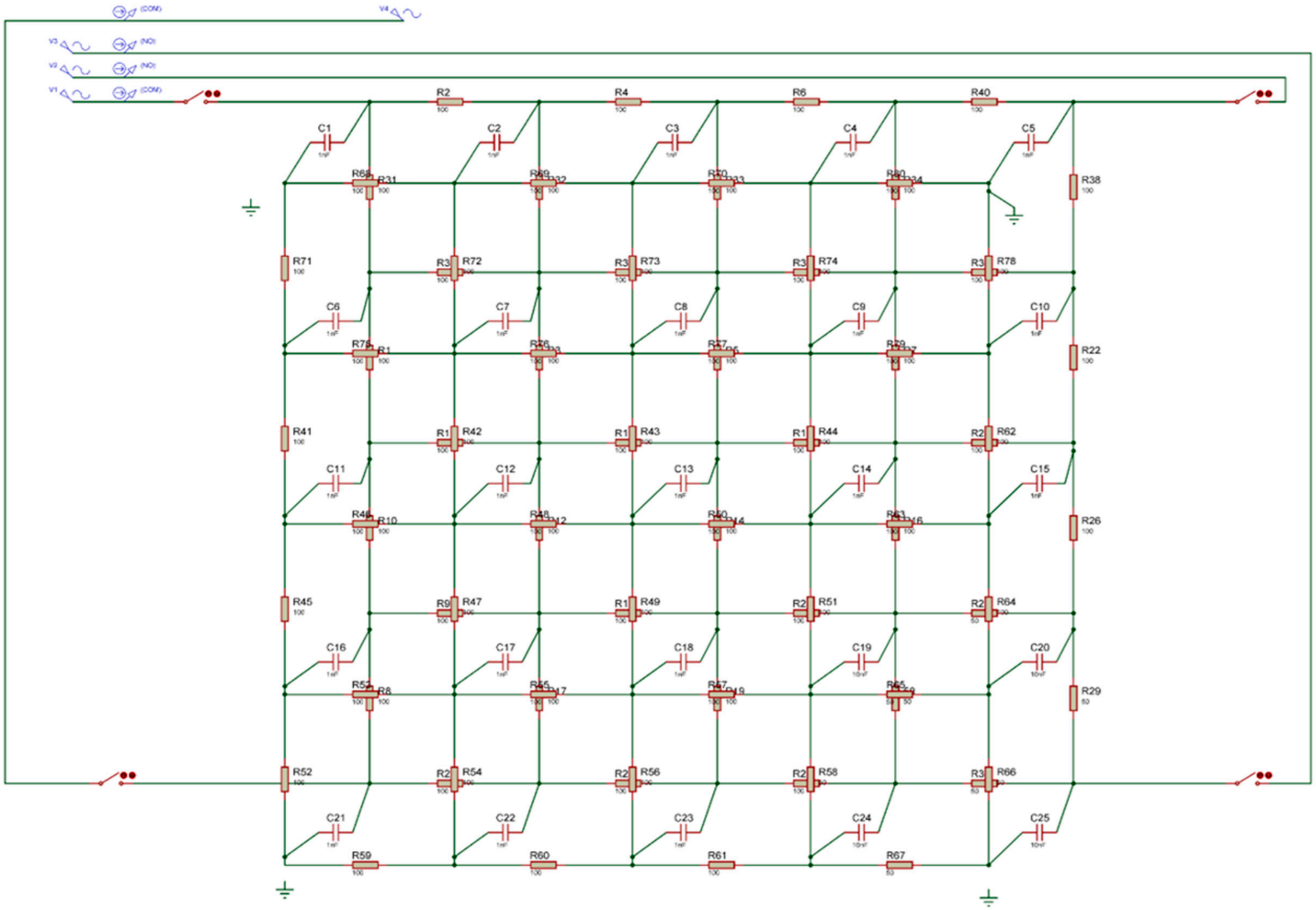

Figure 5. The equivalent circuit of the sensor structure (extended version of the equivalent circuit given in Figure 2d). The simulation is realized for a 5V, 200 Hz signal. The pressure is applied to Location #16 (labeled in Figure 4c in the article).
